# Right Coronary Artery Originating from the Left: Do Not Miss the Diagnosis!

**DOI:** 10.1155/2018/1210791

**Published:** 2018-03-20

**Authors:** Sedat Türkoğlu, Serkan Ünlü, Gülten Aydoğdu Taçoy, Murat Özdemir

**Affiliations:** Department of Cardiology, Gazi University School of Medicine, Ankara, Turkey

## Abstract

**Objective:**

Left circumflex (LCx) artery originating from the right coronary arterial (RCA) system has been reported as the most common form of anomalous origination of a coronary artery from the opposite sinus (ACAOS). However, some studies claim that RCA originating from the left coronary sinus (LCS) is the most frequent form. The aim of this study was to determine the most common type of ACAOS in a single center.

**Materials and Methods:**

The database of the catheterization laboratory was retrospectively searched. All patients who were performed coronary angiography between 1999 and 2006 were included to registry. All examinations were carefully analyzed to determine the most frequent type of ACAOS.

**Results:**

We detected ACAOS in 35 cases (16 RCA originating from the LCS, 13 LCx from the RCS or the RCA, and 6 others) out of 5165 coronary angiograms. The most common form was RCA originating from LCS. Moreover, we revealed that 5 cases with RCA originating from the LCS were previously misdiagnosed and not reported as a coronary anomaly.

**Conclusions:**

RCA originating from the LCS was the most common form of ACAOS in our registry. The high change of misdiagnosis or underreporting of this anomaly could have biased the true prevalence.

## 1. Introduction

Anomalous origination of a coronary artery from the opposite sinus (ACAOS) represents the most common form of coronary artery anomalies in adults [1–3]. ACAOS locating between the aorta and the pulmonary artery could cause serious consequences, including cardiovascular death [4–7]. The left circumflex (LCx) artery originating from either the proximal right coronary artery (RCA) or the right coronary sinus (RCS) has been reported as the most frequent ACAOS in most studies [1–3, 8–15]. In addition, it is usually accepted as a “benign” anomaly [16] since the course of this anomaly is always retroaortic [1, 14, 17, 18]. On the other hand, RCA originating from the left coronary sinus (LCS) usually follows an interarterial course [1, 14, 17, 18]; thus, it may be considered as a life-threatening anomaly [16]. The prevalence of coronary anomalies could show regional differences. Moreover, the experience of the operators can highly influence the diagnosis. Therefore, we planned the present study to investigate the prevalence of coronary artery anomalies in patients who were performed coronary angiography in our center.

## 2. Materials and Methods

A consecutive series of 5165 patients undergoing their first coronary angiography during the period between February 1999 and August 2006 were investigated to create our registry. Original coronary angiography reports were first screened with respect to the final diagnosis of ACAOS. After first screening, all angiographic images of 5165 patients were reevaluated with giving particular attention to the presence of the coronary anomalies. All angiograms were evaluated by three independent investigators by using previously published guides [19, 20]. In case of any difference in opinion, a consensus was reached among readers. Patients with complex congenital heart disease and who were performed recurrent coronary angiographies during the indicated time span were excluded from this analysis. Clinical data concerning participants, such as age, gender, history of hypertension, hypercholesterolemia, diabetes mellitus, smoking status, and family history, were carefully noted from records.

## 3. Results

**Figure 1:**
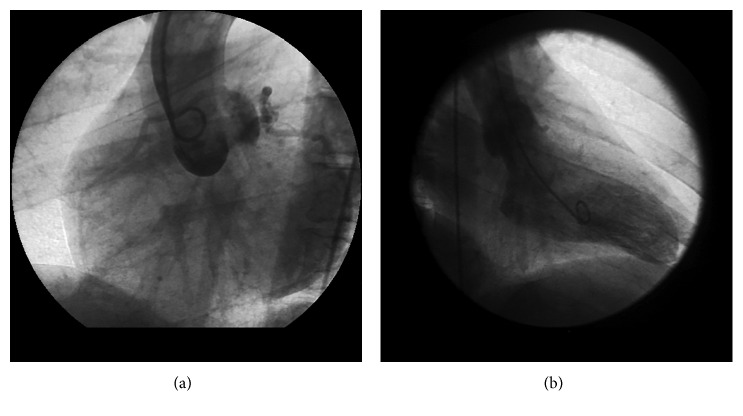
A case which was misdiagnosed as normal coronary arteries. However, with aortography in the left anterior oblique projection (a), it can be recognized that RCA originates from the LCS; with ventriculography in the right anterior oblique projection (b), a mark is seen on the location anterior to aorta, showing interarterial course.

**Figure 2:**
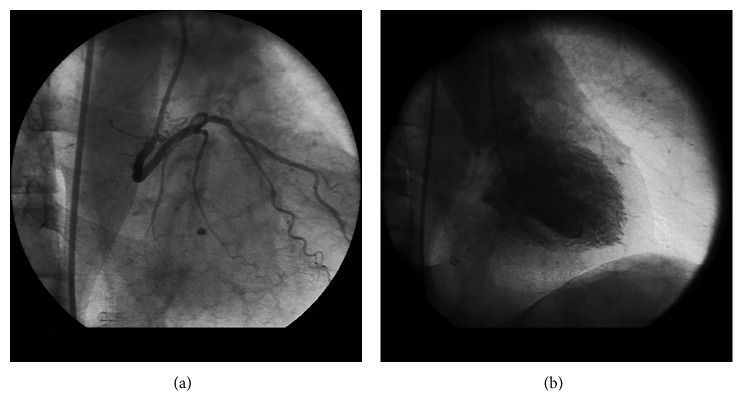
Right coronary sinus injection in the right anterior oblique view: anomalous left circumflex coronary artery is visualized to arise from the right coronary sinus and courses posterior to the aorta (a). Ventriculography in the right anterior oblique view shows a mark posterior to the aorta confirming retroaortic course (b).

The study included 5165 patients (3465 males and 1700 females; mean age 58.6 years with a range of 17 to 90 years) who underwent a first coronary angiography in our hospital between February 1999 and August 2006 for various indications. A total of 35 patients (24 males and 11 females; mean age 60 years with a range of 21 to 80 years) were found to have ACAOS (prevalence 0.68%). The most common anomaly was RCA taking origin from the LCS (16 cases, prevalence = 0.31%) (Tables 1 and 2). In all those cases, anomalous RCA followed an interarterial course (Figure 1). The second most common anomaly was LCx originating from either the RCS or the proximal RCA (13 cases, prevalence = 0.25%). In all these cases, the anomalous LCx followed a retroaortic pathway (Figure 2). The next most prevalent anomaly was the left main coronary artery (LMCA) originating from the RCS. There were 3 patients with this condition. The anomalous LMCA followed the septal pathway in two patients, and its course was retroaortic for the other patient.

We determined 3 cases with ACAOS who had a different pathology from mentioned most common three anomalies. In one of these patients, which was published as a case report previously [21], LCx originated from the proximal RCA and followed the retroaortic course, and the left anterior descending (LAD) originated from the RCS by following the pathway on the anterior free wall. In another patient, LAD originated from the RCS and followed the septal course. Lastly, one patient had an anomalous RCA originating from the midregion of LAD. In that case, the anomalous RCA followed the course on the anterior free wall.

## 4. Discussion

ACAOSs represent the most common form of coronary artery anomalies in adults [1–3]. In most of the large conventional angiographic studies in which coronary anomalies were investigated, anomalous origin of the LCx from the RCS or proximal RCA is the most common type of ACAOS having a prevalence of 0.17–0.45% [1–3, 8–15]. RCA originating from the LCS shows a higher variability of prevalence in these registries. In most of the studies, including the world's largest registry on coronary anomalies [1], it has been reported as the second most common coronary artery anomaly after the anomaly of LCx originating from the proximal RCA or RCS [2, 3, 8–10, 15]. But other studies reveal conflicting results. The anomaly of RCA originating from the LCS was not even reported once in two studies which were performed in Hungary [22] and England [13] by enrolling 7694 and 9153 patients, respectively. On the other hand, two other studies note this anomaly as the most frequent type of ACAOS [17, 18]. These conflicting findings have usually been attributed to racial and regional differences. In our study, we revealed that in 5 of 16 patients with RCA originating from the LCS anomaly, the diagnosis had been missed in the original coronary angiography report, whereas all other types of ACAOS were diagnosed correctly as an anomaly. If we had only screened our original reporting database, we would have erroneously ended up reporting that the LCx originating from the RCS or proximal RCA was the most common form of ACAOS, which was not the in fact case. Therefore, we claim that the diagnosis of the cases with RCA originating from the LCS anomaly might have been misdiagnosed/unnoticed in retrospective angiographic series; thus, its currently known prevalence may have been falsely underreported. In this retrospective study, we avoided such an error by reevaluating all coronary angiograms irrespective of the original diagnosis reported at the time of coronary angiography. Moreover, to our knowledge, the only prospectively designed angiographic study found that RCA originating from the LCS is more common than LCx originating from the RCS [23], which is consistent with our findings and results. In addition, all of the recent studies, except one [24], that used computed tomography (CT) angiography, found RCA originating from the LCS as the most common form of ACAOS [25–31]. This indicates that our findings strongly reflect the real clinical prevalence of coronary anomalies, since CT angiography was shown to be superior to conventional angiography in delineating the origin and the pathway of the anomalous coronary arteries by providing three-dimensional construction of the data [32–34]. So we claim that some cases of RCA originating from the LCS anomaly may have been missed in conventional angiography studies which possibly resulted in lower prevalence of the mentioned anomaly than it should be. On the other hand, diagnosis of anomalous origin of the LCx from the RCS or proximal RCA with conventional angiography is clearly more uncomplicated, leading to a correct diagnosis even performed by a novice clinician.

The anatomy of the coronary arteries that originate from the opposite sinus and follow an interarterial course could result in unwanted consequences and even mortality [4–7]. Although RCA taking origin from the LCS could follow an intraseptal or retroaortic course [1, 30], this anomaly usually follows an interarterial path reaching to 88–100% of the cases [1, 14, 17, 18, 24, 27, 28, 30]. Therefore, correct diagnosis carries high importance since misdiagnosis most likely results in fatal consequences. Our study suggests that some cases with this anomaly may easily be missed to correctly diagnose at the time of coronary angiography. To avoid such a mistake and its potential fatal complications, in cases where the RCA cannot be cannulated selectively, this anomaly must be kept in mind and every effort should be spent to disclose its presence.

The main deficiency of our study is the lack of clinical follow-up information of patients with coronary anomalies due to retrospective nature of the study. Moreover, no data from additional imaging modalities, including CT angiography, could be used as a result for the same reason.

## 5. Conclusions

In conclusion, the most common ACAOS was RCA originating from the LCS. Underreporting the prevalence of this anomaly might have biased the true prevalence in the literature.

## Figures and Tables

**Table 1:** Prevalence of anomalous origination of a coronary artery from the opposite sinus in 5165 patients who underwent a first coronary angiography.

Anomaly	Number of patients	Prevalence (%)
RCA from LCS	16	0.31
LCx from RCS or proximal RCA	13	0.25
LMCA from RCS	3	0.06
LCx from RCA and LAD from RCS	1	0.02
LAD from RCS	1	0.02
RCA from LAD	1	0.02

LAD: left anterior descending; LMCA: left main coronary artery; LCS: left coronary sinus; LCx: left circumflex; RCA: right coronary artery; RCS: right coronary sinus.

**Table 2:** Baseline patient characteristics.

	All ACAOS (*n*=35)	RCA from LCS (*n*=16)	Others^∗^ (*n*=19)
Age (years), mean ± SD	60 ± 13.7	55.9 ± 17.1	63.6 ± 9.2
Male, *n* (%)	24 (69)	9 (56)	15 (79)
Diabetes mellitus, *n* (%)	4 (11)	2 (13)	2 (11)
Current or prior smoker, *n* (%)	20 (57)	9 (56)	11 (58)
Hypertension, *n* (%)	19 (54)	9 (56)	10 (53)
Hyperlipidemia, *n* (%)	10 (29)	7 (44)	3 (16)
Family history early CAD, *n* (%)	3 (9)	3 (19)	3 (16)

ACAOS: anomalous origination of a coronary artery from the opposite sinus; CAD: coronary artery disease; LAD: left anterior descending; LMCA: left main coronary artery; LCS: left coronary sinus; LCx: left circumflex; RCA: right coronary artery; RCS: right coronary sinus; ^∗^others include LCx from RCS or proximal RCA (*n*=13), LMCA from RCS (*n*=3), LCx from RCA and LAD from RCS (*n*=1), LAD from RCS (*n*=1), and RCA from LAD (*n*=1).
